# Blood pressure and site-specific cancer mortality: evidence from the original Whitehall study

**DOI:** 10.1038/sj.bjc.6601255

**Published:** 2003-09-30

**Authors:** G D Batty, M J Shipley, M G Marmot, G Davey Smith

**Affiliations:** 1 Department of Social Medicine, Institute of Public Health, University of Copenhagen, Blegdamsvej 3, DK-2200 Copenhagen N, Denmark; 2 Department of Epidemiology and Public Health, University College London, 1-19 Torrington Place, London WC1E 6BT, UK; 3 Division of Epidemiology, Department of Social Medicine, University of Bristol, Canynge Hall, Whiteladies Road, Bristol, BS8 2PR, UK

**Keywords:** blood pressure; men; epidemiology; neoplasm; whitehall

## Abstract

Studies relating blood pressure to cancer risk have some shortcomings and have revealed inconsistent findings. In 17 498 middle-aged London-based government employees we related systolic and diastolic blood pressure recorded at baseline examination (1967–1970) to the risk of cancer mortality risk at 13 anatomical sites 25 years later. Following adjustment for potential confounding and mediating factors, inverse associations between blood pressure and mortality due to leukaemia and cancer of the pancreas (diastolic only) were seen. Blood pressure was also positively related to cancer of the liver and rectum (diastolic only). The statistically significant blood pressure–cancer associations seen in this large-scale prospective investigation offering high power were scarce and of sufficiently small magnitude as to be attributable to chance or confounding.

Examination of the predictive value of blood pressure for later mortality has a long research pedigree: among the earliest investigations of risk were those undertaken by the US life insurance industry over four decades ago (Society of Actuaries, 1959). The finding in this study of a positive relation between blood pressure and all-cause mortality was later demonstrated for cardiovascular disease (CVD) (Kannel *et al*, 1986; Miura *et al*, 2001). In these investigations, the magnitude of the relation of blood pressure with CVD is typically greater than that between blood pressure and total mortality; similarly, in randomised trials, anti-hypertensive drug treatment lowers CVD incidence but not necessarily total mortality (Neal *et al*, 2000). This suggests that the association between blood pressure and non-CVD causes of death – including cancer – is null or possibly even negative.

The potential link between blood pressure and cancer risk was first examined by de Waard and Baanders-van Halewijn (1974) in a cohort of women, and Dyer *et al* (1975) in a cohort of men, where the finding of a positive relation prompted a series of subsequent investigations (Svardsudd and Tibblin, 1979; Raynor *et al*, 1981; Khaw and Barrett-Connor, 1984; Goldbourt *et al*, 1986; Fraser *et al*, 1990; Grove *et al*, 1991; Filipovsky *et al*, 1993; Hole *et al*, 1993; Wannamethee and Shaper, 1996). Collectively (Grossman *et al*, 2002), there is some evidence that blood pressure is related to an elevated risk of all-cancers combined and renal cancer. Owing to a general paucity of data, however, the relationship between blood pressure and other cancer sites is inconclusive and justifies further examination. Further, existing data are hampered by methodological issues, including reverse causality, lack of adjustment for some potential confounding factors, and inadequate statistical power owing to a small number of cases.

Against this background of inconsistent findings and study limitations we examined the relation of blood pressure to cancer risk at 13 anatomical sites using a 25-year mortality surveillance of 18 403 male government workers in the original Whitehall study, a prospective investigation.

## MATERIALS AND METHODS

In the Whitehall study, data were collected on 18 403 non-industrial London-based male government employees aged from 40 to 64 years when examined between September 1967 and January 1970, representing a 74% response proportion. Data collection involved the completion of a study questionnaire and participation in a medical examination, both of which have been described in detail elsewhere (Reid *et al*, 1974). In brief, the questionnaire included enquiries regarding civil service employment grade (our indicator of socioeconomic position) (Marmot *et al*, 1978), smoking habits (Reid *et al*, 1976), intermittent claudication (Rose, 1962; Davey Smith *et al*, 1990), angina (Rose, 1962; Rose *et al*, 1977b), chronic bronchitis (Committee on the Aetiology of Chronic Bronchitis, 1965) and use of medication, including blood pressure-lowering drugs. Forced expiratory volume in 1 s (FEV_1_) (adjusted for height (Batty *et al*, 2002)), ischaemia (Rose *et al*, 1977a), fasting plasma cholesterol (Davey Smith *et al*, 1992), 2-h blood glucose (Jarrett *et al*, 1986), height (Leon *et al*, 1995), and weight (Jarrett *et al*, 1982) were determined using standardised protocols.

### Assessment of blood pressure

Having been seated while the questionnaire was checked, blood pressure was recorded from a single reading taken from the left arm by a trained observer (Rose, 1965) using a calibrated London School of Hygiene sphygmomanometer (Rose *et al*, 1964). Systolic blood pressure was recorded at the first appearance of the Korotkoff sounds; diastolic pressure was recorded at both their muffling (phase IV) and disappearance (phase V).

### Ascertainment of cancer mortality

The records of 18 260 men (99.2% of subjects) were traced and ‘flagged’, using the procedures of the Office for National Statistics (Southport), until 31 January 1995. Death certificates were coded according to the eighth revision of the International Classification of Diseases (ICD) (Anon., 1967). The category of all malignant neoplasms (ICD 140-208) – referred to here as ‘all-cancers’ – was divided into 13 individual sites: oesophagus (ICD 150); stomach (ICD 151); colon (ICD 153); rectum (ICD 154); liver (ICD 155–156); pancreas (ICD 157); trachea, bronchus and lung (ICD 162 – referred to as ‘lung cancer’); prostate (ICD 185); bladder (ICD 188); kidney (ICD 189); brain (ICD 191); lymphoma (ICD 200–203); and leukaemia (ICD 204–207).

### Data analyses

Our analyses are based on 17 498 men without missing data and exclude 26 men for whom the cause of death was unknown. In analyses of baseline characteristics according to levels of blood pressure, the prevalence of these characteristics were adjusted for age (5-year age groups) by the direct standardisation method. Trends in these proportions were tested for statistical significance using the Mantel–Haenszel test. For baseline characteristics expressed as continuous variables, least-squares means were used to present the age-adjusted means, and tests for trend across blood pressure groups were computed by fitting a linear trend term.

Cox's (Cox, 1972) proportional hazards regression model was used to compute rate ratios and accompanying confidence intervals for the association between components of blood pressure and each mortality outcome. In these analyses we divided each component of blood pressure into tertiles, using the lowest tertile as the referent category. We also fitted systolic and diastolic blood pressure as continuous variables, using these coefficients to test for linear trend and compute the rate ratio per 10 mmHg increase in each blood pressure component.

These models were initially adjusted for age and then for other potential confounding (employment grade, smoking status, blood pressure-lowering medication, marital status, disease at study entry) and mediating (body mass index, triceps skinfold thickness, FEV_1_, cholesterol) variables. Age, cholesterol, body mass index, triceps skinfold thickness, and height-adjusted FEV_1_ were fitted as continuous variables, whereas employment grade (five levels), marital status (four), blood pressure-lowering medication (two), smoking status (four) (with additional adjustment for the number of cigarettes smoked per day in current smokers) and disease at entry (two) were fitted as categorical variables. Disease at entry was defined as a positive response to enquiries regarding one or more of the following at study entry: unexplained weight loss in the preceding year, bronchitis, diabetes (based on self-report and blood glucose levels), ischaemia (based on self-report and ECG trace), intermittent claudication, dyspnoea, or care of a general practitioner for raised blood pressure or heart problems.

For some malignancies (e.g., renal cancer), tumour presence may elevate blood pressure so generating a positive blood pressure–cancer association. To address this issue of reverse causality, we excluded deaths in the first 10 years of follow-up and repeated our analyses. In doing so, we reasoned that a large proportion of deaths attributable to cancer, if present at study induction, would have occurred within this time frame (Brenner, 2002).

## RESULTS

The relation of components of blood pressure to baseline physical characteristics is presented in Table 1

**Table 1:** Association between components of blood pressure and baseline physical characteristics^a^

	**Systolic blood pressure (mmHg)**				**Diastolic blood pressure (mmHg)**			
	**Tertile 1**	**Tertile 2**	**Tertile 3**		**Tertile 1**	**Tertile 2**	**Tertile 3**	
	**(⩽125)**	**(126–142)**	**(⩾143)**	**P-trend**	**(⩽77)**	**(78–88)**	**(⩾89)**	**P-trend**
Number	5833	5947	5718	—	5573	6096	5829	—
								
	*Mean (s.e.)*							
Age	50.2 (0.08)	51.2 (0.08)	53.3 (0.08)	<0.001	51.0 (0.08)	51.3 (0.08)	52.3 (0.08)	<0.001
Tricep skinfold thickness (mm)	42.3 (0.21)	44.9 (0.21)	47.0 (0.21)	<0.001	41.4 (0.21)	44.6 (0.20)	48.0 (0.21)	<0.001
Body mass index (kg m^−2^)	24.0 (0.04)	24.8 (0.04)	25.5 (0.04)	<0.001	23.7 (0.04)	24.7 (0.04)	25.8 (0.04)	<0.001
Plasma cholesterol (mmol l^−1^)	5.07 (0.02)	5.12 (0.02)	5.14 (0.02)	0.002	5.03 (0.02)	5.13 (0.02)	5.16 (0.02)	<0.001
FEV_1_^b^ (l s^−1^)	3.15 (0.01)	3.15 (0.01)	3.09 (0.01)	<0.001	3.14 (0.01)	3.14 (0.01)	3.11 (0.01)	0.004
								
	*Percent (s.e.)*							
Current cigarette smoker	44.2 (0.67)	41.0 (0.64)	39.0 (0.66)	<0.001	47.5 (0.67)	40.7 (0.63)	36.3 (0.63)	<0.001
Low work grade	23.1 (0.57)	22.8 (0.53)	25.2 (0.55)	0.007	23.8 (0.56)	22.7 (0.52)	25.0 (0.54)	0.12
No partner	11.7 (0.43)	11.4 (0.41)	12.3 (0.45)	0.31	11.6 (0.43)	11.5 (0.41)	12.5 (0.44)	0.18
Disease at study entry	21.0 (0.55)	21.2 (0.53)	26.7 (0.59)	<0.001	21.1 (0.55)	21.4 (0.52)	26.3 (0.57)	<0.001
Blood pressure-lowering medication	0.08 (0.03)	0.59 (0.10)	3.72 (0.25)	<0.001	0.11 (0.04)	0.74 (0.11)	3.59 (0.24)	<0.001

a Adjusted for age (age is unadjusted).

b FEV_1_=forced expiratory volume in 1 s adjusted for height.

. Cigarette smoking was inversely related to blood pressure levels, such that the lowest prevalence was seen in the higher tertiles of each blood pressure component. For all other characteristics, however, the most favourable levels of each was apparent in the lower blood pressure groups with statistical significance seen for most, if not all relationships.

In this cohort there were 7460 deaths over 25 years of follow-up. A total of 2226 deaths were ascribed to all-cancers, the most common site being that of the lung (30% of cancer deaths). Rate ratios for the association of systolic and diastolic blood pressure with mortality from all cancers and site-specific cancers are depicted in Tables 2

**Table 2:** Rate ratios (95% confidence intervals) for systolic and diastolic blood pressure in relation to site-specific cancer mortality

	**All-cancers**		**Lung**		**Oesophagus**		**Stomach**		**Pancreas**		**Colon**		**Rectum**	
	**(2226 deaths)**		**(658 deaths)**		**(77 deaths)**		**(157 deaths)**		**(107 deaths)**		**(198 deaths)**		**(71 deaths)**	
	**Age-adjusted**	**Multiply- adjusted^a^**	**Age-adjusted**	**Multiply-adjusted**	**Age-adjusted**	**Multiply-adjusted**	**Age-adjusted**	**Multiply-adjusted**	**Age-adjusted**	**Multiply-adjusted**	**Age-adjusted**	**Multiply-adjusted**	**Age-adjusted**	**Multiply-adjusted**
*Systolic blood pressure (mmHg)*														
Tertile 3	0.96	0.98	0.76	0.82	1.23	1.14	1.21	1.18	0.73	0.72	0.81	0.78	1.27	1.20
(⩾143)	(0.87, 1.07)	(0.89, 1.10)	(0.62, 0.92)	(0.67, 1.00)	(0.70, 2.13)	(0.64, 2.01)	(0.82, 1.77)	(0.80, 1.76)	(0.45, 1.18)	(0.44, 1.19)	(0.57, 1.14)	(0.55, 1.12)	(0.71, 2.27)	(0.66, 2.17)
Tertile 2	0.94	0.97	0.86	0.94	1.00	0.98	0.90	0.91	0.86	0.87	0.80	0.80	1.03	1.00
(126–142)	(0.85, 1.05)	(0.88, 1.07)	(0.72, 1.04)	(0.78, 1.13)	(0.57, 1.75)	(0.56, 1.72)	(0.60, 1.35)	(0.61, 1.37)	(0.55, 1.35)	(0.55, 1.36)	(0.57, 1.12)	(0.57, 1.12)	(0.57, 1.87)	(0.55, 1.81)
Tertile 1	1.0	1.0	1.0	1.0	1.0	1.0	1.0	1.0	1.0	1.0	1.0	1.0	1.0	1.0
(⩽125)														
Per 10 mmHg	1.00	1.00	0.95	0.97	1.00	0.98	1.06	1.05	0.94	0.94	0.96	0.95	1.04	1.03
increase	(0.97, 1.02)	(0.98, 1.02)	(0.92, 0.99)	(0.93, 1.01)	(0.90, 1.12)	(0.88, 1.11)	(0.98, 1.14)	(0.98, 1.14)	(0.85, 1.04)	(0.85, 1.04)	(0.89, 1.03)	(0.88, 1.02)	(0.93, 1.16)	(0.91, 1.16)
*P* for trend	0.62	0.89	0.011	0.15	0.94	0.79	0.15	0.18	0.21	0.22	0.24	0.17	0.54	0.65
														
*Diastolic blood pressure (mmHg)*														
Tertile 3	0.91	0.99	0.63	0.79	1.30	1.24	1.11	1.14	0.63	0.64	0.98	0.94	1.88	1.84
(⩾89)	(0.82, 1.01)	(0.89, 1.10)	(0.52, 0.76)	(0.65, 0.97)	(0.71, 2.38)	(0.66, 2.32)	(0.76, 1.62)	(0.76, 1.70)	(0.39, 1.02)	(0.39, 1.05)	(0.70, 1.38)	(0.65, 1.34)	(1.06, 3.34)	(1.01, 3.34)
Tertile 2	0.86	0.92	0.72	0.85	1.64	1.66	0.90	0.94	0.72	0.74	0.88	0.86	0.96	0.95
(78–88)	(0.78, 0.96)	(0.83, 1.02)	(0.60, 0.86)	(0.71, 1.02)	(0.94, 2.88)	(0.94, 2.93)	(0.61, 1.34)	(0.64, 1.40)	(0.46, 1.13)	(0.47, 1.17)	(0.63, 1.24)	(0.61, 1.22)	(0.50, 1.83)	(0.50, 1.82)
Tertile 1	1.0	1.0	1.0	1.0	1.0	1.0	1.0	1.0	1.0	1.0	1.0	1.0	1.0	1.0
(⩽77)														
Per 10 mmHg	0.99	1.02	0.90	0.98	1.01	0.99	1.03	1.04	0.86	0.86	0.96	0.95	1.19	1.19
increase	(0.96, 1.02)	(0.99, 1.06)	(0.85, 0.95)	(0.92, 1.04)	(0.85, 1.20)	(0.83, 1.18)	(0.92, 1.16)	(0.92, 1.18)	(0.74, 1.00)	(0.73, 1.01)	(0.87, 1.07)	(0.85, 1.06)	(1.01, 1.40)	(1.00, 1.41)
*P* for trend	0.50	0.24	0.0004	0.46	0.88	0.89	0.61	0.52	0.05	0.06	0.51	0.36	0.04	0.05

a Adjustment for confounding (age, employment grade, smoking status, blood pressure-lowering medication, marital status, disease at study entry) and mediating (body mass index, triceps skinfold thickness, FEV_1_, cholesterol) variables.

and 3

**Table 3:** Rate ratios (95% confidence intervals) for systolic and diastolic blood pressure in relation to site-specific cancer mortality

	**Prostate**		**Bladder**		**Brain**		**Leukaemia**		**Lymphoma**		**Liver**		**Kidney**	
	**(252 deaths)**		**(92 deaths)**		**(47 deaths)**		**(76 deaths)**		**(107 deaths)**		**(38 deaths)**		**(47 deaths)**	
	**Age-adjusted**	**Multiply-adjusted^a^**	**Age-adjusted**	**Multiply-adjusted**	**Age-adjusted**	**Multiply-adjusted**	**Age-adjusted**	**Multiply-adjusted**	**Age-adjusted**	**Multiply-adjusted**	**Age-adjusted**	**Multiply-adjusted**	**Age-adjusted**	**Multiply-adjusted**
*Systolic blood pressure (mmHg)*														
Tertile 3	0.96	1.05	1.07	1.18	1.10	1.07	0.69	0.66	1.07	1.03	6.12	5.35	1.42	1.26
(⩾143)	(0.71,1.31)	(0.76,1.44)	(0.62, 1.85)	(0.67, 2.06)	(0.55, 2.18)	(0.53, 2.15)	(0.40, 1.19)	(0.38, 1.17)	(0.66, 1.76)	(0.62, 1.72)	(2.07, 18.1)	(1.78, 16.1)	(0.72, 2.82)	(0.62, 2.59)
Tertile 2	0.97	0.99	1.44	1.53	0.75	0.73	0.61	0.60	1.25	1.24	3.55	3.54	0.77	0.78
(126–142)	(0.72,1.32)	(0.73,1.35)	(0.87, 2.39)	(0.92, 2.54)	(0.37, 1.55)	(0.35, 1.51)	(0.35, 1.06)	(0.35, 1.04)	(0.79, 1.97)	(0.78, 1.96)	(1.17, 10.8)	(1.16, 10.8)	(0.36, 1.66)	(0.36, 1.67)
Tertile 1	1.0	1.0	1.0	1.0	1.0	1.0	1.0	1.0	1.0	1.0	1.0	1.0	1.0	1.0
(⩽125)														
														
Per 10 mmHg	1.00	1.02	1.04	1.06	0.99	0.99	0.91	0.90	1.03	1.02	1.23	1.19	1.10	1.06
increase	(0.93,1.06)	(0.95,1.08)	(0.94, 1.14)	(0.96, 1.18)	(0.86, 1.15)	(0.85, 1.16)	(0.80, 1.02)	(0.79, 1.01)	(0.93, 1.13)	(0.92, 1.12)	(1.07, 1.41)	(1.03, 1.37)	(0.96, 1.25)	(0.92, 1.21)
*P* for trend	0.93	0.61	0.48	0.25	0.94	0.92	0.10	0.08	0.61	0.74	0.003	0.02	0.17	0.45
														
*Diastolic blood pressure (mmHg)*														
Tertile 3	1.05	1.15	0.81	0.95	0.51	0.45	0.57	0.54	1.19	1.20	3.44	3.03	1.21	1.12
(⩾89)	(0.77,1.44)	(0.83,1.59)	(0.48, 1.36)	(0.54, 1.64)	(0.23, 1.13)	(0.20, 1.04)	(0.33, 0.98)	(0.30, 0.96)	(0.76, 1.87)	(0.75, 1.93)	(1.26, 9.40)	(1.07, 8.59)	(0.62, 2.37)	(0.54, 2.29)
Tertile 2	1.12	1.15	1.05	1.15	1.02	0.95	0.54	0.53	0.77	0.80	3.17	3.21	0.69	0.70
(78–88)	(0.83,1.51)	(0.85,1.56)	(0.65, 1.70)	(0.71, 1.87)	(0.54, 1.93)	(0.50, 1.82)	(0.32, 0.98)	(0.31, 0.92)	(0.47, 1.25)	(0.49, 1.30)	(1.17. 8.59)	(1.17, 8.77)	(0.33, 1.46)	(0.33, 1.48)
Tertile 1	1.0	1.0	1.0	1.0	1.0	1.0	1.0	1.0	1.0	1.0	1.0	1.0	1.0	1.0
(⩽77)														
Per 10 mmHg	1.00	1.03	1.01	1.08	1.00	0.99	0.83	0.81	1.10	1.11	1.42	1.38	1.15	1.09
increase	(0.91,1.10)	(0.93,1.14)	(0.87, 1.18)	(0.92, 1.27)	(0.81, 1.24)	(0.78, 1.25)	(0.69, 0.99)	(0.67, 0.98)	(0.96, 1.27)	(0.96, 1.29)	(1.16, 1.73)	(1.10, 1.73)	(0.94, 1.41)	(0.88, 1.36)
*P* for trend	0.99	0.62	0.87	0.36	0.99	0.93	0.04	0.03	0.17	0.17	0.001	0.005	0.19	0.42

a Adjustment for confounding (age, employment grade, smoking status, blood pressure-lowering medication, marital status, disease at study entry) and mediating (body mass index, triceps skinfold thickness, FEV_1_, cholesterol) variables.

. Given that the results of models adjusting for confounding variables only, and confounding and mediating variables combined, were very similar, we only present the latter as ‘multiple’ adjustment.

Both systolic and diastolic blood pressure were essentially unrelated to total cancer mortality. In age-adjusted analyses, inverse associations of cancer of the lung, and pancreas, and leukaemia with blood pressure were seen at conventional levels of statistical significance. Following multiple adjustment, the relationship with lung cancer was lost. Blood pressure was positively related to cancer of the rectum (diastolic only) and liver; confidence intervals latter, confidence intervals were wide owing to the low number of deaths. In the analyses in which we excluded deaths occurring in the first 10 years of follow-up, a positive association between diastolic, but not systolic, blood pressure and lymphoma emerged (rate ratio, 95% confidence interval for 10 mmHg increase in blood pressure; 1.16, 0.99–1.36, *P* [trend]=0.07).

## DISCUSSION

In this large, prospective cohort study, blood pressure was related to few of the cancer end points under investigation at conventional levels of statistical significance. Thus, an inverse association between blood pressure and mortality due to pancreatic cancer (diastolic only) and leukaemia was seen after adjustment for potential confounding and mediating factors. After exclusion of deaths in the first decade of follow-up, the relation for leukaemia was lost. Both systolic and diastolic blood pressure were positively related to cancer of the liver, rectum and, after exclusion of early deaths, lymph nodes.

### Confounding and reverse causality

The interpretation of the findings of several studies of the blood pressure–cancer relation is complicated by issues of confounding and reverse causality. The effect of potentially important confounding variables such as social circumstances, alcohol consumption and anti-hypertensive medications is rarely explored. Anti-hypertensive medications have been linked to increased risk of malignancy in some (Armstrong *et al*, 1974; Fletcher *et al*, 1993) but not all (Dyer *et al*, 1975; Raynor *et al*, 1981) studies, raising the possibility that it is not high blood pressure or hypertension *per se* that is generating the association. However, few investigators have data on medication and blood pressure levels to allow them to explore this issue. In the present study, the already weak blood pressure–cancer associations seen after controlling for age were generally little altered when we added other factors to our regression models. While information on those men taking medication for high blood pressure was available, the prevalence at baseline was very low (1.5%), and there were insufficient cancer deaths in this group to examine either the predictive value of blood pressure-lowering drugs for cancer risk or explore their potential confounding effect.

We observed a positive relation between blood pressure and liver cancer. Many of these malignancies would have been attributable to alcohol-induced cirrhosis of the liver, a precipitating condition. Both systolic and diastolic blood pressure were positively and incrementally related to liver cirrhosis deaths rates (rate ratio, 95% confidence interval for 10 mmHg increase in systolic blood pressure; 1.21, 1.04–1.41, *P* [trend]=0.01). This suggests that the positive blood pressure–liver cancer relation may be confounded by alcohol consumption; however, we only have data on alcohol intake for a 10% subsample of the cohort – too few to investigate this possibility. We are unaware of any other studies to have examined the link between blood pressure and liver cancer, a scarcity that is also apparent for pancreatic cancer and leukaemia.

In a small study of renal cancer patients, blood pressure levels normalised following tumour removal (Lee, 1971); while in others (Kirchner *et al*, 1976; Melman *et al*, 1977) the prevalence of renal cell carcinoma in hypertension patients exceeded that in the general population. It is therefore plausible that, for this type of neoplasm, which has been consistently linked with high blood pressure levels (Grossman *et al*, 2002), the presence of tumours may raise blood pressure, rather than the reverse, so generating a positive blood pressure–cancer association (Buck and Donner, 1987). In the present analyses, risk of mortality from this cancer was also elevated in the high blood pressure groups, but not significantly so. The high number of cancer deaths in the present study afforded us the opportunity of exploring this issue in more depth. On excluding deaths from renal cancer occurring in the first 10 years of follow-up, this association was eliminated suggesting that reverse causality may be the underlying explanation. This is also a likely justification for the blood pressure–leukaemia association.

In none of our analyses was either component of blood pressure related to mortality from cancer of combined sites. Findings from other studies are inconsistent with positive (Svardsudd and Tibblin, 1979; Goldbourt *et al*, 1986; Fraser *et al*, 1990; Filipovsky *et al*, 1993) and null (Grove *et al*, 1991; Hole *et al*, 1993) associations reported. Owing to its high incidence, lung cancer is the site-specific malignancy most commonly examined in relation to blood pressure. In the present study, there was a weak, inverse relation of systolic and diastolic blood pressure with lung cancer, a finding that is, again, supported by some (Khaw and Barrett-Connor, 1984), but not all (Svardsudd and Tibblin, 1979; Goldbourt *et al*, 1986), investigators.

To summarize, of the associations observed, blood pressure and liver cancer is likely to be due to confounding by alcohol consumption, while blood pressure and leukaemia is probably attributable to reverse causality, as described. For the remaining two statistically significant relationships, rectal and pancreatic cancer were essentially null for systolic blood pressure and barely reached statistical significance for diastolic pressure. Given that we related 17 outcomes (the cancers plus total mortality, CHD, and stroke) to two components of blood pressure, it is highly plausible that these latter two associations are attributable to the play of chance, or, because of their small magnitude, confounding by unmeasured factors, residual confounding by measured factors, or both.

### Limitations of the present study

In the present study, systolic and diastolic blood pressure were measured on a single occasion, raising concerns about validity. We addressed this issue by relating these exposures to coronary heart disease and stroke mortality, both of which have been shown to be positively associated with systolic and diastolic blood pressure in a pooling of individual level data for one million subjects (Lewington *et al*, 2002). In the present analyses, as in earlier follow-ups of the same cohort (Lichtenstein *et al*, 1985), the relationship between blood pressure and both these end points was positive, incremental, and strong, suggesting our blood pressure data have predictive validity. It is also likely that our blood pressure measurements would have changed over 25 years of follow-up. Given that these fluctuations would have been non-differential with respect to the outcome of interest, any reported associations would have been weaker than the true, underlying relationships (Armstrong *et al*, 1994).

In conclusion, in this large-scale prospective investigation of male government employees offering a large number of cases, an inverse association between blood pressure and mortality from cancer of the pancreas and leukaemia was seen, while blood pressure was positively related to liver and rectal cancer risk. That these relationships were few among the many analyses we conducted, and of small magnitude, suggests that they are likely to be due to chance and/or confounding rather than being real effects.
